# Pre-Formulation Studies: Physicochemical Characteristics and In Vitro Release Kinetics of Insulin from Selected Hydrogels †

**DOI:** 10.3390/pharmaceutics13081215

**Published:** 2021-08-06

**Authors:** Aneta Ostróżka-Cieślik, Małgorzata Maciążek-Jurczyk, Jadwiga Pożycka, Barbara Dolińska

**Affiliations:** 1 Department of Pharmaceutical Technology, Faculty of Pharmaceutical Sciences in Sosnowiec, Medical University of Silesia, Kasztanowa 3, 41-200 Sosnowiec, Poland; bdolinska@sum.edu.pl; 2 Department of Physical Pharmacy, Faculty of Pharmaceutical Sciences in Sosnowiec, Medical University of Silesia, Jagiellońska 4, 41-200 Sosnowiec, Poland; mmaciazek@sum.edu.pl (M.M.-J.); jpozycka@sum.edu.pl (J.P.); 3 “Biochefa” Pharmaceutical Research and Production Plant, Kasztanowa 3, 41-200 Sosnowiec, Poland

**Keywords:** hydrogel, insulin, in vitro drug release study, rheology, texture analysis, spectroscopic methods

## Abstract

Insulin loaded to the polymer network of hydrogels may affect the speed and the quality of wound healing in diabetic patients. The aim of our research was to develop a formulation of insulin that could be applied to the skin. We chose hydrogels commonly used for pharmaceutical compounding, which can provide a form of therapy available to every patient. We prepared different gel formulations using Carbopol^®^ Ultrez^TM^ 10, Carbopol^®^ Ultrez^TM^ 30, methyl cellulose, and glycerin ointment. The hormone concentration was 1 mg/g of the hydrogel. We assessed the influence of model hydrogels on the pharmaceutical availability of insulin in vitro, and we examined the rheological and the texture parameters of the prepared formulations. Based on spectroscopic methods, we evaluated the influence of model hydrogels on secondary and tertiary structures of insulin. The analysis of rheograms showed that hydrogels are typical of shear-thinning non-Newtonian thixotropic fluids. Insulin release from the formulations occurs in a prolonged manner, providing a longer duration of action of the hormone. The stability of insulin in hydrogels was confirmed. The presence of model hydrogel carriers affects the secondary and the tertiary structures of insulin. The obtained results indicate that hydrogels are promising carriers in the treatment of diabetic foot ulcers. The most effective treatment can be achieved with a methyl cellulose-based insulin preparation.

## 1. Introduction

Insulin is a peptide hormone secreted in a pulsatile manner by the beta cells of the pancreatic islets. Under normal conditions, insulin secretion is two-phase. The first phase is quick and lasts up to 10 min. During this time, the insulin accumulated in the secretory granules is released. The second, in turn, is longer, lasts from 2 to 3 h, and reflects the ability of the pancreas to produce insulin [1]. The human form of insulin has a molecular weight of 5.8 kDa and consists of two chains: A/alpha (consisting of 21 amino acid residues) and B/beta (containing 30 amino acid residues). The chains are connected by two disulphide bridges located between Cys-A7 and Cys-B7 as well as Cys-A20 and Cys-B19. In the A chain, there is a third disulphide bond linking Cys-A6 and Cys-A11 [2,3]. Insulin plays an important role in maintaining whole-body homeostasis. It stimulates DNA replication and participates in the regulation of carbohydrate metabolism and the synthesis of proteins and glycogen. It participates in lipid metabolism, ion and amino acid transport, cell proliferation and differentiation, and nitric oxide synthesis. It affects tissues by connecting with the insulin membrane receptor, which is a glycoprotein made up of two alpha subunits and two beta subunits. The alpha subunits are located on the surface of cell membranes of tissues and organs and are connected by disulfide bonds with the beta subunits, which in turn pass through the cell membrane into the cells [4,5]. The beneficial effect of insulin in the wound healing process was observed already at the beginning of the 20th century. Surgeons confirmed its positive effect in postoperative wound healing in patients with and without diabetes. Insulin receptors are present in keratinocytes and epidermal fibroblasts. Insulin stimulates proliferation, migration, and secretion of keratinocytes, endothelial cells, and fibroblasts. Stimulation of keratinocyte migration is dose- and time-dependent in an insulin receptor-dependent but EGF/EGF-R-independent manner. It is suggested that this is associated with increased expression of the integrin α3β1 in keratinocytes and an increase in the levels of LN332 (Laminin 332). Insulin may act as a chemoattractant and mitogenic agent for cells that are involved in wound healing [6,7]. The insulin receptors IRS-1 (insulin receptor substrate-1), IRS-2 (insulin receptor substrate-2), ERK (extracellular signal-regulated protein kinase), and Akt (serine-threonine kinase) are significantly expressed in the damaged wound vs. the intact skin, indicating a potential role for the insulin signaling pathway in the wound repair process. Insulin shows anti-inflammatory capacity by increasing the production of chemokines IL-4, IL-13, and Il-10 and decreasing the production of IFN-Υ. It also shows inactivation of the inflammatory pathway mediated by TNF-α during fat metabolism [8].

Mutations in the insulin receptor can lead to the development of insulin resistance and, consequently, to the development of serious diseases, including diabetes. One of the most serious complications of diabetes is diabetic foot ulceration. It is estimated that 15% of diabetic patients develop foot ulcers as a result of peripheral neuropathy or ischemia. These types of skin lesions are particularly prone to infections and gangrene, which can often lead to necrosis and, consequently, limb amputation [9]. In recent years, significant research was devoted to the development of effective treatments and prevention of diabetic foot ulcers. Many therapeutic models were proposed, including larval therapy and use of stem cells and growth factors [10,11]. However, the cost of these therapies is high, and their safety is debatable. It is suggested that topical insulin is the optimal alternative. However, its effective application to the skin requires the development of a vehicle that effectively delivers the hormone to the wound bed. A promising direction is the development of a semi-solid form of insulin, e.g., as adhesive films or hydrogel. The use of bioadhesive hydrogels may increase the prolonging residence time of insulin at the site of application and reduce the frequency of administration [7]. Carriers that deliver insulin in its free form use the location of the insulin receptor located on the plasma membrane [12].

A review of the literature on the subject indicates that insulin affects the regeneration and the reconstruction of damaged skin [13,14,15,16,17]. Goenka et al. [13] used an insulin preparation to treat patients with diabetic ulcers. Wounds were sprayed with four units (0.1 mL) of human soluble insulin (Actrapid) in 1 mL of saline (0.9%) for every 10 cm^2^ of the wound area. Insulin affected regeneration and reconstruction of damaged skin without affecting the blood glucose level in diabetic people. Topical insulin was safe and effective without systemic side effects. Besson et al. [14] investigated the effectiveness of insulin (dose: 50 IU) in a complex with 2-hydroxypropyl-β-cyclodextrin (HPβCD) in the erythrocyte cell model. The released insulin modulated the reepithelialization process, stimulating cell proliferation and migration of keratinocytes, boosting matrix remodeling. Lima et al. [15] developed a cream, a carrier for topical insulin application (patent number BRPI0705370 B1), the effectiveness of which was tested in the male Wistar rat model with induced diabetes. The effective insulin concentration was 0.5 U/100 g cream. They found a significant improvement in wound healing compared to the control rats. They observed the reversal of defective insulin signal transduction and increased expression of eNOS (endothelial nitric oxide synthase), VEGF (vascular epidermal growth factor), and SDF-1α (stromal cell-derived factor-1α). The same authors, in another paper using an analogous research model [16], observed an improvement in reepithelialization, tissue granulation, and increased collagen deposition. Zhao et al. [17] developed a CSPBA/PVA/OHC-PEG-CHO hydrogel (CSPBA, phenylboronic modified chitosan; PVA, poly (vinyl alcohol); OHC-PEGCHO, benzaldehyde-capped poly (ethylene glycol)) containing 0.5 mL of bovine insulin. The effectiveness of the formulation was tested using the L929 mouse fibroblast cell model. They found that the developed insulin carrier promoted neovascularization and collagen deposition in the process of diabetic wound healing. Sridharan et al. [18] conducted a meta-analysis of randomized controlled trials on the efficacy of insulin in wound healing. They concluded that topical insulin application reduces the risk of hypoglycemia vs. other routes of administration.

Recently, a great deal of research was done on “smart hydrogels”. Hydrogels were used to construct glucose biosensors. The protein was placed in the pores of the membrane, or the protein was chemically bound to the membrane [19]. Polymer hydrogels swell or contract in response to changes in glucose level, allowing delivery of optimal insulin doses. The release of insulin from the glucose-responsive hydrogels occurs through the membrane, or the hydrogel acts as a reservoir [20]. Matsumoto et al. [21] developed a synthetic “smart gel” which exhibits an artificial pancreas-like function in vivo. They used phenylboronic acid (PBA) to self-regulate insulin delivery.

The obtained research results are promising, and the need to perform further analyses aimed at the development of an effective, safe, and economically accessible treatment method is up-to-date and of great social importance.

The aim of our research was to develop a formulation of insulin that could be applied to the skin. We chose hydrogels commonly used for pharmaceutical compounding, which can provide a form of therapy available to every patient, i.e., Carbopol^®^ UltrezTM 10, Carbopol^®^ UltrezTM 30 hydrogel based on methyl cellulose and glycerol ointment. We selected carriers that are promising for clinical applications [22,23]. Their advantages include simple production technology, low financial costs associated with their preparation, high efficiency, high biocompatibility, low sensitizing potential, and an esthetic and modern form of application. They provide a moist environment in the wound area, which affects the speed and the quality of the healing process. They also show high stability of the physical form, which allows the active substance to be introduced into the gel in the form of a solution or a suspension without the risk of sedimentation [23]. Hydrogels are carriers that protect and deliver peptides due to their physical properties. They influence the maintenance of a high local concentration of peptides over a long period of time [22]. We assessed the influence of model hydrogels on the pharmaceutical availability of insulin in vitro, and we examined the physicochemical properties of the prepared formulations.

## 2. Materials and Methods

### 2.1. Materials

Insulin Actrapid Penfill 100 j.m./mL (the other ingredients were zinc chloride, glycerol, metacresol, sodium hydroxide, hydrochloric acid, and water for injections) was purchased from Novo Nordisk (Bagsværd, Denmark). Carbopol^®^ Ultrez^TM^ 10 (INCI: Carbomer) and Carbopol^®^ Ultrez^TM^ 30 (INCI: Carbomer) were purchased from Lubrizol (Cleveland, OH, USA). Carbopol^®^ Ultrez™ 10 and Carbopol^®^ Ultrez^TM^ 30 were cross-linked polyacrylic acid polymers. Methyl cellulose was purchased from Fluka Chemie, Buchs, Switzerland. Wheat starch was purchased from PPH Galfarm (Kraków, Poland). Glycerol was purchased from Pharma Cosmetic, Poland. Ethanol was purchased from Alpinus Chemia, Poland. Triethanolamine (TEA) was purchased from Chempur, Śląskie, Poland. Sodium chloride was purchased from POCH, Gliwice, Poland. All chemicals and solvents used for the study were of analytical grade.

### 2.2. Preparation of Hydrogels

**Figure 1:**
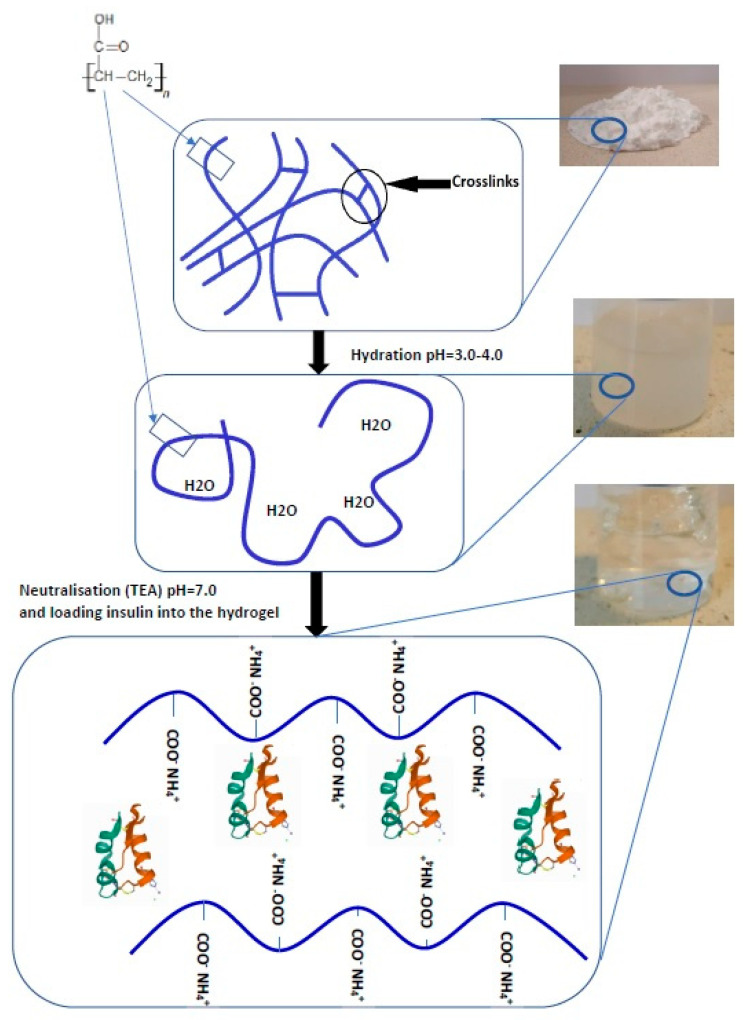
The schematic shows the chemical structure of Carbopol and the mechanism of thickening. Carbopol in its dry form—tightly coiled acidic molecules. Hydration—the molecules begin to hydrate and partially uncoil. Neutralization with triethanolamine (TEA)—conversion of the acidic Carbopol polymer to salt loading insulin into the hydrogels. The structure of insulin was obtained from the Protein Data Bank (PDB, http://www.rcsb.org, accessed on 25 July 2021) under code PDB 3E7Y [26].

Hydrogel preparations were prepared er the requirements of the Polish Pharmacopoeia XI [24], according to the formula provided in Table 1. Triethanolamine was used for neutralizing carboxyl groups and spatial cross-linking of Carbopol^®^ Ultrez™ 10 (H1) and Carbopol^®^ Ultrez™ 30 (H2) [25]. The formulation pH was neutral. Figure 1 shows the chemical structure of Carbopol and the mechanism of thickening. The methyl cellulose-based hydrogel (H3) was prepared by hot dispersing of the polymer in water. The glycerol ointment (H4) was prepared based on the formula provided in the Polish Pharmacopoeia XI [24]. The wheat starch was mixed with water, and glycerol was added in portions (adding small amounts repeatedly) while stirring. The whole was heated in a water bath until it became homogenous. After cooling, ethanol was added. Insulin (INS) was introduced into the prepared H1–H4 hydrogels. The active ingredient solution was mixed with a hydrogel base by mechanical stirring for 15 min [24]. The hormone concentration was 1 mg/g of hydrogel each time. All chemicals and solvents used for the preparation of hydrogels were of analytical grade.

### 2.3. Stability Studies

Hydrogels stability was tested by the stability test of biotechnological/biological products under conditions specified by ICH, Q5C: 25 ± 1 °C and 5 ± 3 °C. The samples were stored for 4 weeks [27]. Hydrogels were controlled by visual examination, drug content, pH, and viscosity. The pH of hydrogels was tested with the SevenCompact^TM^ S210 device (Mettler Toledo, Switzerland) using the InLab^®^Expert Pro–ISM electrode with a solid polymeric electrolyte (Mettler Toledo, Switzerland). The measurement accuracy was ±0.01 pH. The analysis for color, homogeneity, and phase separation was performed in daylight in glass beakers placed against a white matte background [24]. The amount of insulin was determined by spectrophotometry using a CECIL apparatus (CE 3021, Cambridge, United Kingdom) at a wavelength λ = 271 nm. Rotational rheometry was used for viscosity tests using the Lamy RM 200 Touch laboratory rheometer (Lamy Rheology Instruments, Champagne au Mont d’Or, France) equipped with the MK-CP 2445 measuring system and the CP-1 Plus laboratory thermostat. All prepared formulations were found to be stable. API content was within the acceptable limit of 90% of the initial value [28].

### 2.4. Insulin Release In Vitro

The pharmaceutical availability of insulin from the hydrogels was tested with the USP apparatus 2 (vessel volumes 200 mL) at 100 rpm (Erweka DT600, Husenstamm, Germany) using the Enhancer Cell™ with a surface area of 3.80 cm^2^ (Erweka, Husenstamm, Germany). A regenerated cellulose dialysis membrane Spectra/Por^®^ 2 was used (MWCO of 12–14 kDa; Spectrum Laboratories, Inc., California, CA, United States) [29,30]. Enhancer cells were filled with the appropriate hydrogel in the amount of 1 g. The dialysis membrane was in contact with the hydrogel loaded in the enhancer cell. The acceptor fluid volume was 50 mL (saline, 0.9% NaCl), T = 32 ± 1 °C (normal human skin temperature). The enhancer cell was immersed in the dissolution vessels. The amount of insulin diffusing through the membrane was determined by spectrophotometry using a CECIL apparatus (CE 3021, Cambridge, United Kingdom) at a wavelength λ = 271 nm [31]. The linear dependence of absorbance as a function of the concentration of standard solutions was described by the equation y = 0.453x + 0.0072. The coefficient of determination was R² = 0.999. The precision of the method was assessed positively based on the values of standard deviation, relative standard deviation, and coefficient of variation. All chemicals used for the study were of analytical grade.

### 2.5. Release Kinetics

The kinetics of insulin release in vitro was analyzed using the DDSolver1 software [27]. The release mechanism was analyzed using four different kinetic models, Equations (1)–(4): 

Model Zero order:

(1) $$F=k_{0\;}xt$$

Model First order:

(2) $$F=100\times \left[1-Exp\left(-k_{1}xt\right)\right]$$

Model Higuchi:

(3) $$F=k_{H}xt^{0.5}$$

Model Korsmeyer–Peppas:

(4) $$F=k_{kp}xt^{n}$$

where: *F*, fraction [%] of drug released in time *t*; *k*_0_, zero-order release constant; *k*_1_, first-order release constant; *k_H_*, Higuchi release constant; *k_kp_*, release constant incorporating structural and geometric characteristics of the drug-dosage form; *n*, diffusional exponent indicating the drug-release mechanism.

### 2.6. Dissolution Profiles Comparison

The release profiles of H1-INS, H2-INS, H3-INS, and H4-INS hydrogels were compared using the DDSolver1 software [32]. The method of the factors of similarity *f*2 and difference *f*1 was used. The obtained values were compared with the threshold values. The values of the factors *f*1 Equation (5) and *f*2 Equation (6) were calculated using the following formulas:

Difference factor:

(5) $$f1=\left[\frac{\sum \left|R_{t}-T_{t}\right|}{\sum R_{t}}\right]\;\times \;100$$

Similarity factor:

(6) $$f2=50\times log\left\{{\left[1+\frac{1}{n}\sum {\left(R_{t}-T_{t}\right)}^{2}\right]}^{-0.5}\times \;100\right\}$$

where: *R_t_*, *T_t_*: percentage dissolved of the reference and the test profiles, respectively, at time point *t*; *n*: number of sampling points.

### 2.7. Measurement of Rheological Parameters

Rotational rheometry was used for rheological tests using the Lamy RM 200 Touch laboratory rheometer (Lamy Rheology Instruments, Champagne au Mont d’Or, France) equipped with the MK-CP 2445 measuring system and the CP-1 Plus laboratory thermostat. The measurements were carried out at 25 ± 1 °C (ambient temperature) and 32 ± 1 °C (normal human skin temperature). The flow and the viscosity curves were performed in a controlled shear mode in the range 1.0–100.0 s^−1^ within a time of 100 s. Graphical and mathematical analysis of the obtained results was performed with the Rheometric-P Software. The flow curves of the analyzed hydrogels were plotted. The relations between shear stress and shear rate were analyzed using various rheological models, Equations (7)–(10):

Ostwald-de Waele:

(7) $$\tau =K\times \overset{\cdot }{\gamma }n$$

Herschel–Bulkley:

(8) $$\tau =\tau_{0}+K\times \overset{\cdot }{\gamma }n$$

Bingham:

(9) $$\tau =\tau_{0}+\eta \times \overset{\cdot }{\gamma }$$

Casson:

(10) $$\tau^{0.5}=\tau_{0}^{0.5}+\eta^{0.5}\times {\overset{\cdot }{\gamma }}^{0.5}$$

where: τ, shear stress (Pa); τ_0_, yield stress or yield point; $\overset{\cdot }{\gamma }$, shear rate (s^−1^); K, consistency coefficient (Pa)^1/2^(s)^n^; η, viscosity (Paxs); n, flow behavior index.

### 2.8. Texture Analysis

**Figure 2:**
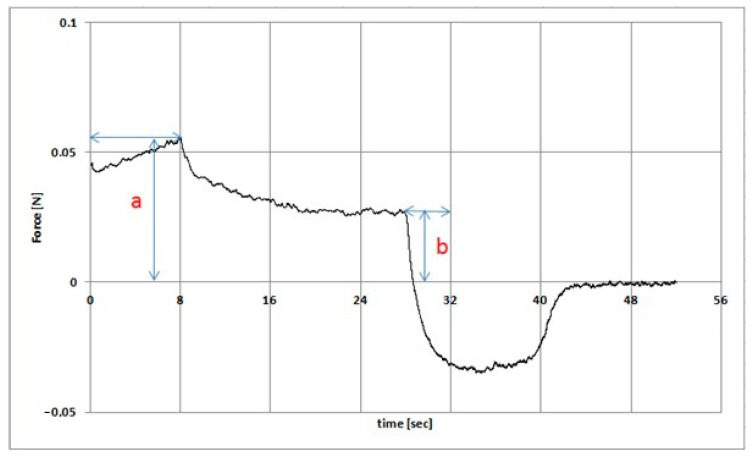
Example of a stress relaxation profile (the symbols are discussed in the text).

The texture analysis of hydrogel samples was performed using the Texture Analyzer TX-700 (Lamy Rheology, Champagne-au-Mont-d’Or, France) equipped with a ½ spherical probe with a diameter of 8 mm. The measurements were made in the compression/relaxation/tension (CRT) and the texture profile analysis (TPA cycle) modes. CRT analysis was performed with the following parameters: down speed 0.5 mm/s; force to start 0.05 N; relaxation time 20 s, wait for position 20 mm (Figure 2). The percentage of relaxation (%R) was determined from the Equation (11):

(11) $$R=\frac{b}{a}\times 100$$

**Figure 3:**
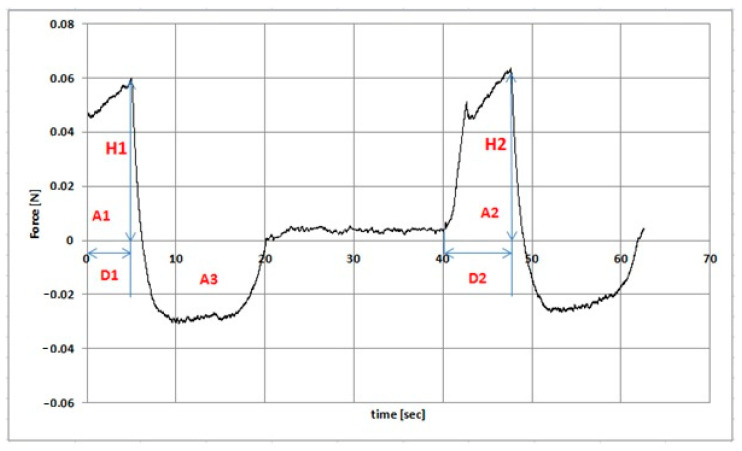
Example of texture profile analysis with parameters (the symbols are discussed in the text).

Parameters *a* and *b* were the forces recorded initially and after 20 s of relaxation [33]. TPA cycle was measured with the following parameters: down speed 1 mm/s; force to start 0.005 N; relaxation time 20 s; wait for position 10 mm (Figure 3). Cohesiveness was determined as the ratio of the area under the curve (force (N) = f (time (s)) in the second cycle A2 to the first cycle A1. Adhesiveness corresponded to the A3 area in Figure 7. Elasticity was determined as the D2/D1 ratio. D2 was the displacement from the starting point of the deformation process in the second cycle to deformation at F2. D1 was the distance from the starting point of the deformation process in the first cycle to the deformation F1. F1 was the maximum force value in the first cycle. F2 was the maximum force value in the second cycle. Hardness corresponded to the maximum force value in the first pressing section [34,35,36]. The measurements were carried out at 25 ± 1 °C and 32 ± 1 °C. The results were recorded and analyzed using the Rheotex software, version TX-UK01/2019.

### 2.9. Circular Dichroism (CD), Emission, and Absorption Spectra Measurements

Far UV-CD spectra of insulin at 1.05·10^−4^ g/cm^3^ concentration were recorded using JASCO J-1500 CD spectropolarimeter equipped with a thermostatic Peltier cell holder with an accuracy of ±0.05 °C. Circular dichroism measurements were made in a nitrogen atmosphere at 25 °C in a 1 mm path length quartz cuvette. Samples were scanned from 200 nm to 250 nm at wavelength intervals of 0.2 nm. Before the calculation of the final ellipticity, CD insulin spectra in both the presence of H_2_O and model hydrogels H1–H4 were corrected by subtraction of solvent spectra measured under identical conditions. Then, using Savitzky and Golay filters method and 17 convolution width, the obtained spectra were smoothed. CD intensity was expressed as mean residue ellipticity at wavelength λ ((θ)mre) according to the Equation (12) [37].

(12) $${\left[\theta \right]}_{\mathrm{mre}}=\frac{\mathrm{MRW}\cdot \theta_{\lambda }}{10\cdot l\cdot c}\;\left[\deg\cdot {\mathrm{cm}}^{2}\cdot {\mathrm{dmol}}^{-1}\right]$$

where: MRW, mean residue weight (MRW_INS_ = 115.87 Da); θ_λ_, observed ellipticity at wavelength λ in deg; l, optical path length in cm; c, insulin concentration in g·cm^−3^.

The fluorescence measurements were recorded using fluorescence spectrophotometer JASCO FP-6500 with quartz cells at 10 mm path length. The accuracy of wavelength was ±1.5 nm. Emission fluorescence spectra of insulin at 1.04 × 10^−5^ g/cm^3^ both in H_2_O and in the presence of model hydrogels H1–H4 were recorded using λ_ex_ 256 nm.

Using a JASCO V-530 spectrophotometer, the absorbance measurements of insulin at 1.04 × 10^−5^ g/cm^3^ both in H_2_O and in the presence of model hydrogels H1–H4 with quartz cells at 10 mm path length in the range between 250 nm and 320 nm were made. The scattering spectrum of solvent was subtracted from all the emission spectra. Both emission and absorption spectra were recorded at 298 K.

In order to study insulin stability, the measurements of insulin in H_2_O immediately after sample preparation and after 72 h were carried out.

### 2.10. Statistical Analysis

The measurements were repeated 3 times. The results are presented as means ± SD. Microsoft Excel 2007 was used for calculations. Significant differences between the means were identified by one-way ANOVA followed by Duncan’s multiple range test. The analysis was performed with the Statistica version 13.1 software (StatSoft, Cracow, Poland). The level of significance was considered at *p* < 0.05.

## 3. Results

**Figure 4:**
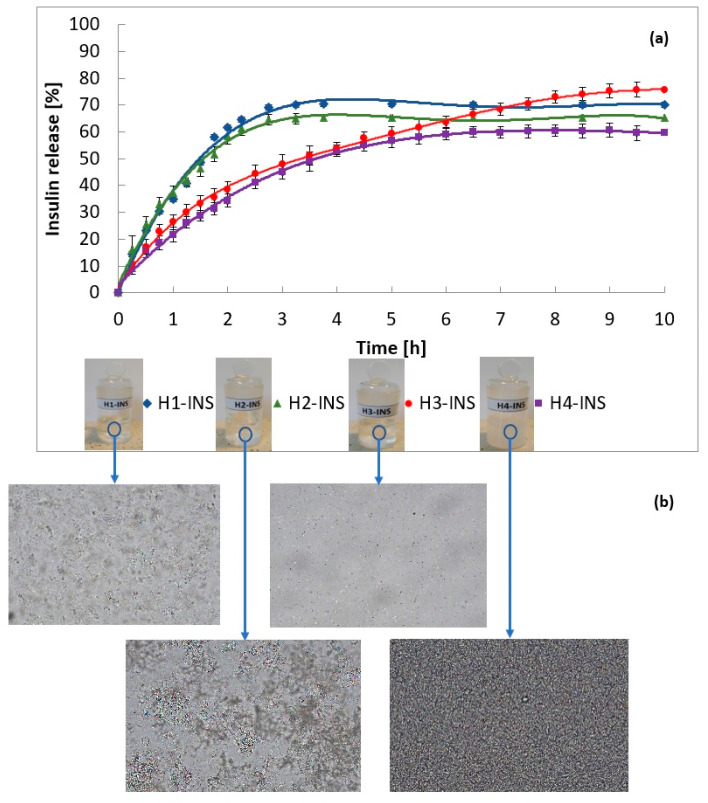
(**a**) Graphical comparison of profiles of insulin release from the tested hydrogels. Each value represents the mean ± SD (*n* = 18). (**b**) Microscope image of hydrogels (100×; polarizing microscope LAB 40, Opta-Tech, Warszawa, Poland).

Four high-quality insulin preparations were prepared, meeting pharmacopoeial requirements and company standards. The amount of insulin released from the hydrogels is shown in Figure 4. In total, 70% of insulin was released from the H1-INS (a hydrogel based on Carbopol^®^ Ultrez^TM^ 10) formulation after 3 h, 65% from H2-INS (a hydrogel based on Carbopol^®^ Ultrez^TM^ 30) after 3 h, 75% from H3-INS (a hydrogel based on methyl cellulose) after 9 h, and 60% from H4-INS (a hydrogel based on glycerol ointment) after 6 h. Insulin release from the H1-INS–H4-INS formulation occurred in a prolonged manner, providing a longer duration of action of the hormone.

The release curve profiles (Figure 4) and determined parameters of fitting the mathematical models to the obtained data (Table 2) indicate that the release of insulin from the H1-INS and the H2-INS preparations followed the Higuchi model, whereas release from H3-INS and H4-INS followed the Korsmeyer–Peppas model. The following were used as the criteria of the model correctness: the coefficient of determination (R^2^) and the Akaike information criterion (AIC). The best model of the active substance release kinetics was characterized by the highest R^2^ and the lowest AIC [38]. According to the Higuchi model, the process of insulin diffusion through pores is a factor that controls the drug release from H1 and H2 hydrogels. The in vitro release profile of the insulin showed a rapid release at 2.5 h and a complete release by 4 h. The amount of released insulin is a function of the square root of time. The profile of insulin release from H3 and H4 hydrogels was consistent with the Korsmeyer–Peppas model. This is a typical model of API release from hydrogel matrices [39]. n_H3-INS_ was 0.399 and n_H4-INS_ was 0.410, therefore, the active substance release occurred following Fick’s diffusion law (*n* < 0.5).

The insulin release rate and the release rate constants varied with the hydrogel type (Table 2). The constant release rates decreased in the following order:

*k_H_ H1-INS* < *k_H_ H2-INS* < *k_kp_ H3-INS* < *k_kp_ H4-INS*

The release profiles of the tested formulations were compared (Table 3). It was found that the profiles of H1-INS vs. H2-INS (*f*1 = 7.69, *f*2 = 68.29) showed similarity. The release profiles were assumed to be similar when *f*1 was in the range of 0–15 and *f*2 was in the range of 50–100. A value of 100 meant that the compared profiles were identical, and a value of 50 represented a 10% difference across all time points [40].

The rheological and the textural parameters are important features of hydrogels indicating their functional properties. They enable one to optimize the process of their production, filling, and storage and to predict the effectiveness of the application and the efficiency of the designed product. Table 4 summarizes the values of dynamic viscosity of the analyzed formulations at the temperatures of 25 °C (preparation storage temperature) and 32 °C (skin surface temperature) obtained at different shear rates. H1-INS, H2-INS, H3-INS, and H4-INS at low shear rates showed high dynamic viscosity, which is characteristic of pseudoplastic liquids. The reason for shear-thinning in polymer solutions may have been the orientation of their polymer chains. At rest, the chaotic movement of the polymer chains prevailed without any specific orientation. In turn, shear forces oriented them in the direction of flow. The parallel arrangement of polymer chains reduced frictional resistance, which translated into a decrease in viscosity. The decrease in viscosity under the influence of shear rate is an important parameter reflecting the effectiveness of topical drug application. The developed formulations allowed the formation of a thin hydrogel layer on the skin, which is associated with their high efficiency. The viscosity of the formulations decreased slightly by 1.0–1.3 times with a temperature increase, which indicates their thermal stability.

**Figure 5:**
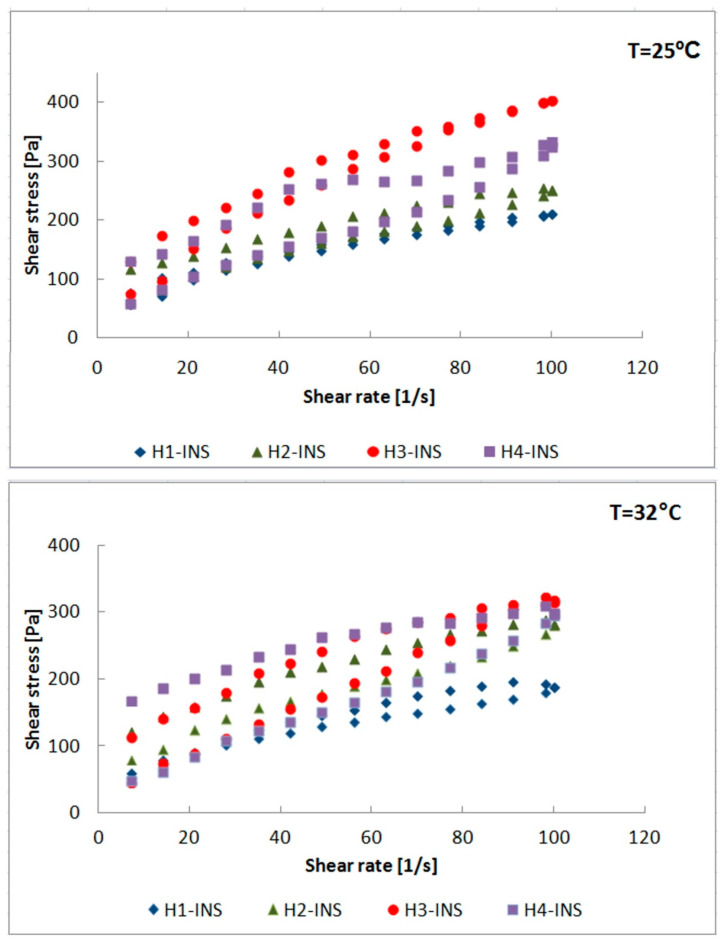
Flow curves hysteresis for H1-INS, H2-INS, H3-INS, and H4-INS (T = 25 °C; T = 32 °C).

The flow curves of the insulin-containing hydrogels were plotted: shear stress (Pa) = f (shear rate (1/s)) at 25 °C and 32 °C (Figure 5). The analysis of rheograms showed that their pattern was typical of shear-thinning non-Newtonian fluids. In order to analyze the changes in the curves, they were approximated with Ostwald-de Waele, Herschel–Bulkley, Bingham, and Casson’s rheological models. The results of calculations of rheological parameters are summarized in Table 5. The value of the flow index *n* < 1 in the Ostwald-de Waele model indicated the shear thinning of fluids, which was confirmed in the Herschel–Bulkley model. The level of fitting with the Herschel–Bulkley model to the experimental data was very high in all cases. The correlation coefficients at T = 25 °C were as follows: R^2^ = 0.994 for H1-INS, R^2^ = 0.997 for H2-INS, R^2^ = 0.998 for H3-INS, R^2^ = 0.993 for H4-INS. In turn, at T = 32 °C, they were: R^2^ = 0.999 for H1-INS, R^2^ = 0.995 for H2-INS, R^2^ = 0.999 for H3-INS, R^2^ = 0.998 for H4-INS. It was found that the consistency coefficient K was the highest for the H1-INS formulation at both temperatures, which shows its highest resistance to deformation depending on the shear rate. The analyzed solutions showed the features of viscoplastic fluids with the yield stress τ_0_. The yield stress τ_0_ is the minimum stress above which the formulation begins to flow. It should be large enough to prevent the hydrogel from flowing out of the container under its own weight (the container positioned upside down) and not too high to ensure adequate spreadability when applied onto the skin. The highest values of the yield stress were found for H2-INS (T = 25 °C, τ_0_ = 34.8 Pa; T = 32 °C, τ_0_ = 34.4 Pa) and H4-INS (T = 25 °C, τ_0_ = 52.0 Pa; T = 32 °C, τ_0_ = 39.6 Pa). Low values of the yield stress were confirmed for H1-INS (T = 25 °C, τ_0_ = 1.6 Pa; T = 32 °C, τ_0_ = 6.2 Pa) and H3-INS (T = 25 °C, τ_0_ = 4.6 Pa; T = 32 °C, τ0 = 14.2 Pa). The obtained results for H1- INS fell within the ranges obtained by Kim et al. (1.59–6.0 Pa [41]), whereas for H2-INS, they corresponded to the values obtained by Islam et al. (28–37 Pa [42]). This suggests that the H1-INS and the H3-INS hydrogels melt on the skin, increasing the surface of insulin diffusion. On the other hand, liquefaction of the structure was lower in the cases of H2-INS and H4-INS, which suggests lower percutaneous diffusion of insulin [43].

Figure 5 shows the flow curves of the analyzed formulations. Under isothermal conditions, by increasing the shear rate and then reducing it to the initial value, hysteresis loops were obtained, which proves the thixotropy of the tested systems. They were characterized by the ability to rebuild the original internal structure after shear cessation after a certain time. Thixotropy is a consequence of breaking and rebuilding the weak physical bonds that hold the internal structure of hydrogels. The thixotropic properties of preparations play an important role in determining their therapeutic efficacy. They influence the retention time of the active substance at the application site and its bioavailability [44,45]. The hysteresis areas of the formulations tested at T = 25°C were as follows: H1-INS 10.66 Pa/s, H2-INS 31.40 Pa/s, H3-INS 27.82 Pa/s, H4-INS 61.54 Pa/s. In turn, at T = 32 °C, they assumed the following values: H1-INS 16.68 Pa/s, H2-INS 39.09 Pa/s, H3-INS 48.94 Pa/s, H4-INS 87.26 Pa/s. The hysteresis area is a measure of the breakdown of the internal structure of hydrogels. It was suggested that a larger area of the hysteresis loop increases the penetration of active substances through the stratum corneum. During topical application, formulations deform and become more fluid, which favors the diffusion of active substances [46,47].

**Figure 6:**
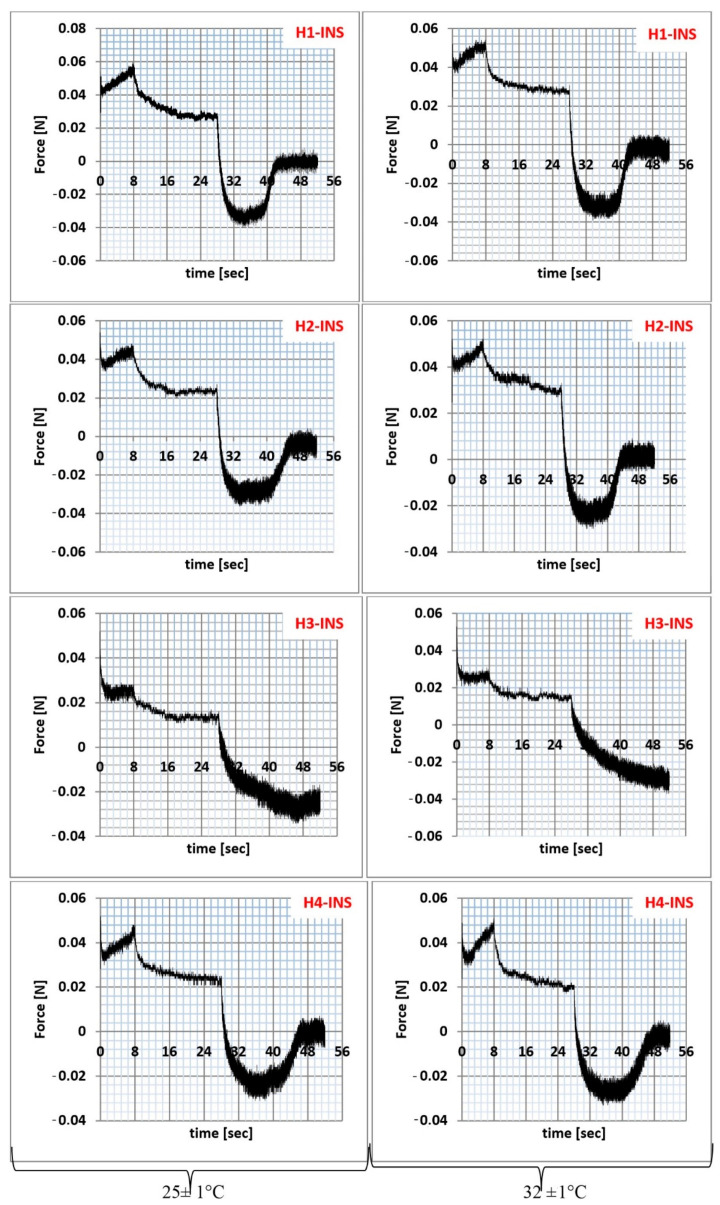
Compression/relaxation/tension (CRT) of hydrogel samples at 25 ± 1 °C and 32 ± 1 °C.

**Figure 7:**
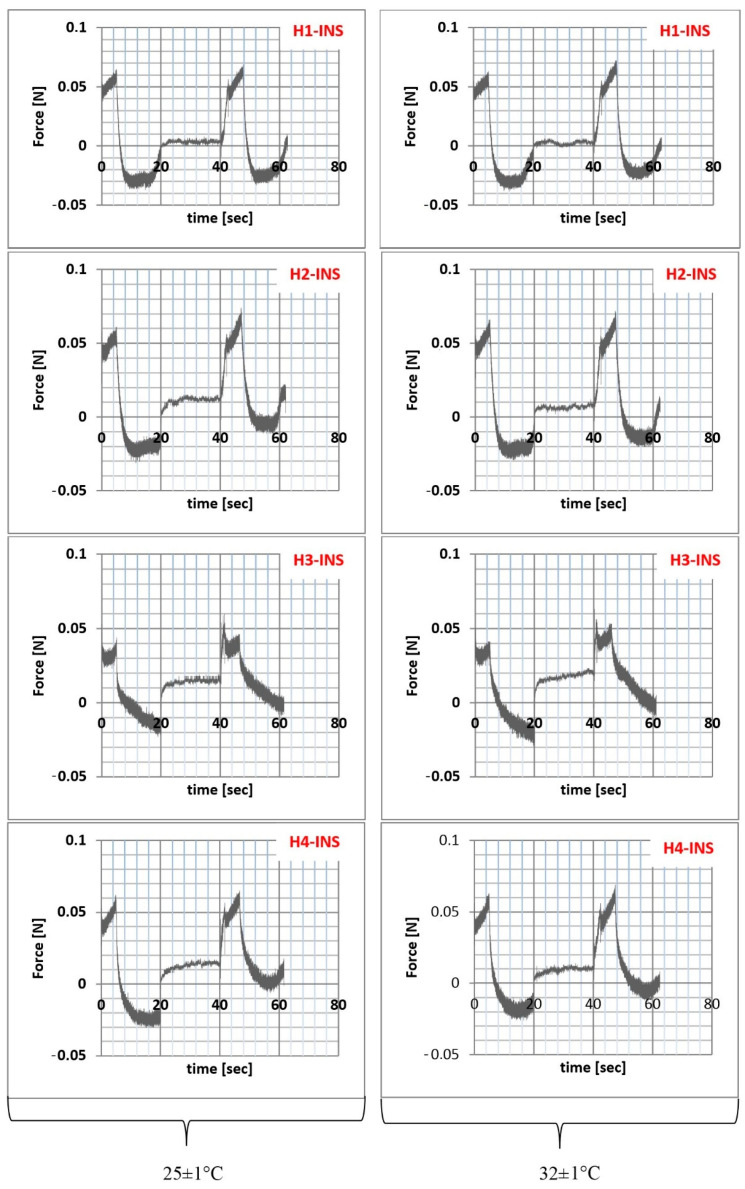
Texture profile analysis (TPA) of hydrogel samples at 25 ± 1 °C and 32 ± 1 °C.

Texture analysis is a penetrometer technique useful in characterizing pharmaceutical preparations in terms of their topical application [47]. It is a relatively simple and quick analytical technique that provides information about the mechanical parameters of hydrogels, i.e., relaxation, hardness, cohesiveness, adhesiveness, and elasticity [48,49]. The results of compression/relaxation/tension (CRT, Figure 6) and texture profile analysis (TPA, Figure 7) are presented graphically and in Table 6.

The percentage of relaxation is determined by the elasticity index. Hydrogels with a higher %R are characterized by lower stiffness [50,51]. The highest %R value was recorded for the H3-INS formulation (72.7%, T = 25 °C; 74.9%, T = 32 °C). The graph patterns in Figure 6 were typical for the analyzed pharmaceutical preparations. Each of them had two peaks corresponding to the first and the second cycles of formulation compression. The peaks in the individual plots varied in height and width. The smaller the differences in peak heights were in the first and the second compression cycles, the higher was the elasticity of the formulation. The lower the hardness one value was, the easier the application to the skin was as well [49]. The lowest value of this parameter was observed for the H3-INS formulation at T = 25°C (0.042 N/m^2^) and T = 32 °C (0.040 N/m^2^). In the other cases, the values were similar (they oscillated around 0.06 N/m^2^). The smallest difference in the heights of the peaks F1 and F2 was found for the H4-INS formulation at T = 25 °C (Δ = 0.003). It is suggested that hydrogels are resistant to deformation, which results from the degree of physical cross-linking of the gelling substance inside the base [52].

Adhesion is an important factor in the production of hydrogels. This parameter is also related to hydrogel retention in skin and bioadhesives [53]. It was found that formulations with high adhesiveness tend to stick to the package surface, making it difficult to use. In our study, the highest values of this parameter were obtained for the formulations H1-INS and H2-INS at T = 25 °C and T = 32 °C, but the differences were statistically insignificant (*p* > 0.05). Cohesiveness is a parameter that determines the degree of damage to the hydrogel structure after its application. It defines the strength and the flexibility of the formulation [49]. Its high value correlates with the reconstruction of the formulation structure. The analyzed hydrogels were characterized by high cohesiveness values (>1), which proves their high cohesiveness and homogeneity. Preparations characterized by both greater cohesiveness and adhesiveness better adhere to the skin surface. Elasticity is the speed at which a deformed sample returns to its original shape after deforming forces are removed. Low values indicate that formulations are sensitive to structural deformation. In the case of the H1-INS hydrogel, the elasticity values at T = 25 °C and T = 32 °C were close to unity, thus it most easily recovered its original shape after deformation [53]. No visual deformation of the structure was observed in any of the analyzed formulations.

**Figure 8:**
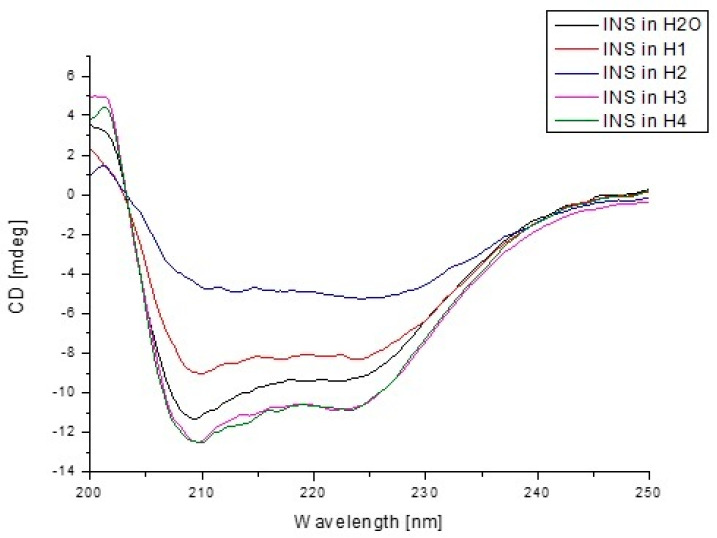
Far-UV circular dichroism spectra of insulin in the presence of H_2_O and model hydrogels.

To assess how the presence of model hydrogels influenced the secondary structure of insulin, circular dichroism spectroscopy was used (Figure 8). The CD spectroscopy is one of the most popular techniques used for the structural characterization of biological samples, e.g., proteins [54]. It is a practical tool for rapid secondary (far-UV CD) and tertiary structure (near-UV CD) determination. Due to the fact that model hydrogels H1, H2, H3, and H4 could be carriers of insulin, in this paper, CD spectroscopy was used to analyze changes in the secondary structure of INS under the influence of hydrogels, and the obtained from CD spectra parameters for H1-INS, H2-INS, H3-INS, and H4-INS were compared with those obtained for INS in H_2_O. The percentage (%) content of the secondary structure elements of INS was obtained using secondary structure estimation program with Reed’s reference models, and data are presented in Table 7.

**Figure 9:**
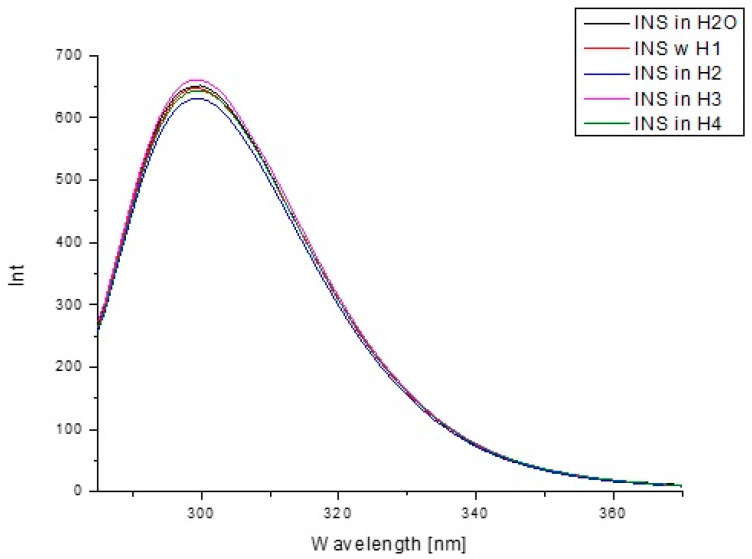
Emission fluorescence spectra of insulin at 1.04 × 10^−5^ g/cm^3^ concentration both in H_2_O and in the presence of model hydrogels H1–H4, λ_ex_ 276 nm.

To determine the influence of hydrogen carriers (H1–H4) on INS tertiary structure, excitation wavelengths λ_ex_ 276 nm (fluorescence emission of tyrosyl residues) were used. Based on the emission fluorescence spectra (Figure 9) of insulin both in H_2_O and in the presence of model hydrogels H1–H4, a decrease in INS fluorescence was observed (Table 8).

**Figure 10:**
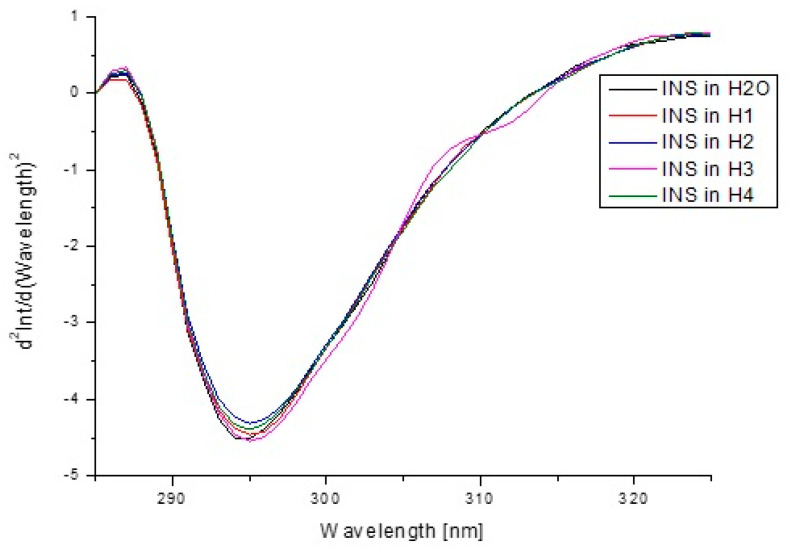
Second derivative emission fluorescence spectra of insulin in H_2_O and in model hydrogels (H1–H4).

The second derivative of fluorescence and absorption spectra is a good method for the evaluation of minor environmental changes in chromophores surroundings caused by the presence of an additional factor. In order to further confirm the effect of H1–H4 carriers on the insulin tertiary structure in the environment of excited at λ_ex_ 276 nm amino acid fluorophores, fluorescence spectra second derivatives were recorded (Figure 10).

**Figure 11:**
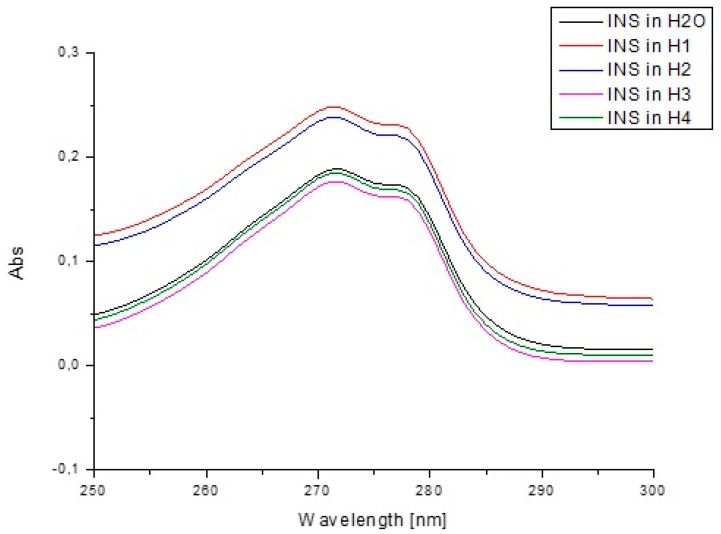
Absorption spectra of insulin at 1.04 × 10^−5^ g/cm^3^ concentration both in H_2_O and in the presence of model hydrogels H1–H4.

**Figure 12:**
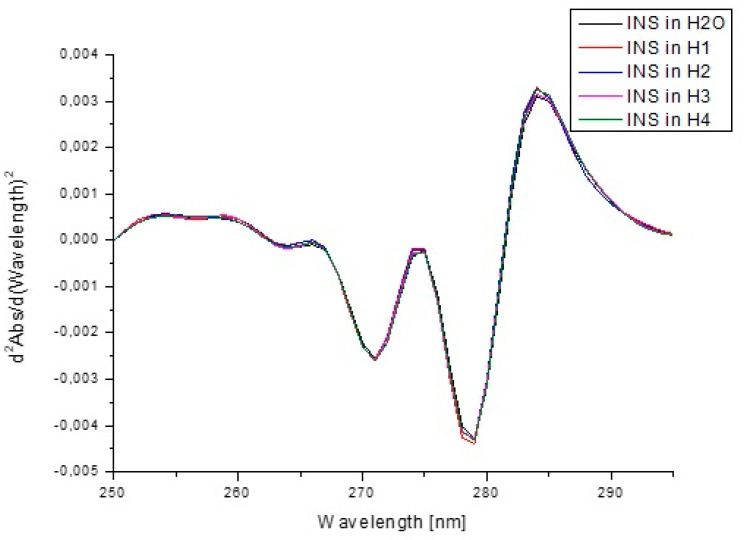
Second derivative absorption spectra of insulin at 1.04 × 10^−5^ g/cm^3^ concentration both in H_2_O and in the presence of model hydrogels H1–H4.

Figure 11 presents the absorption spectra of insulin in H_2_O and model hydrogels, while Figure 12 shows the second derivative of absorption spectra.

**Figure 13:**
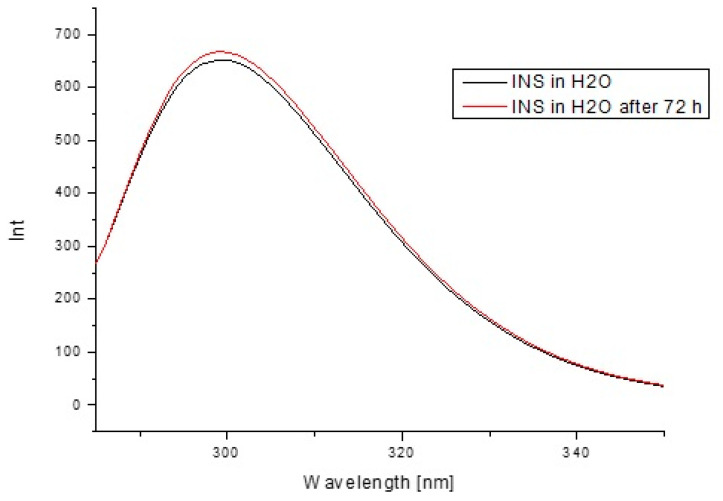
Emission fluorescence spectra of insulin at 1.04 × 10^−5^ g/cm^3^ concentration in H_2_O immediately after sample preparation and after 72 h.

**Figure 14:**
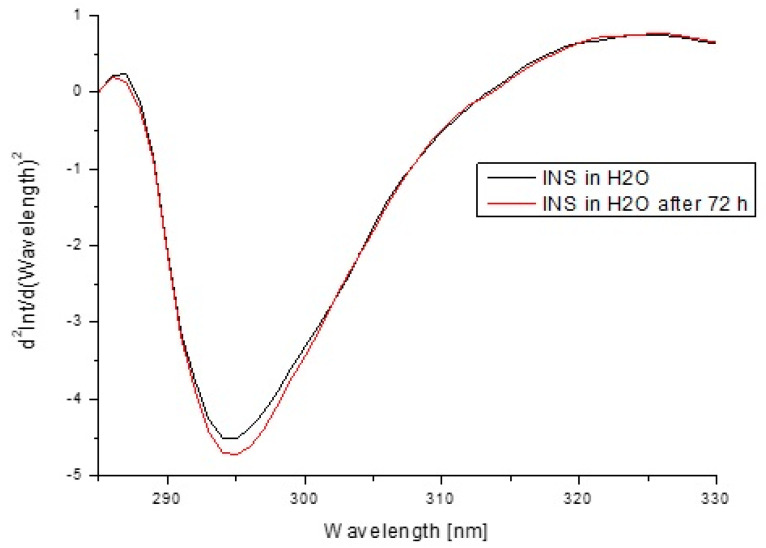
Second derivative emission fluorescence spectra of insulin at 1.04 × 10^−5^ g/cm^3^ concentration in H_2_O immediately after sample preparation and after 72 h.

**Figure 15:**
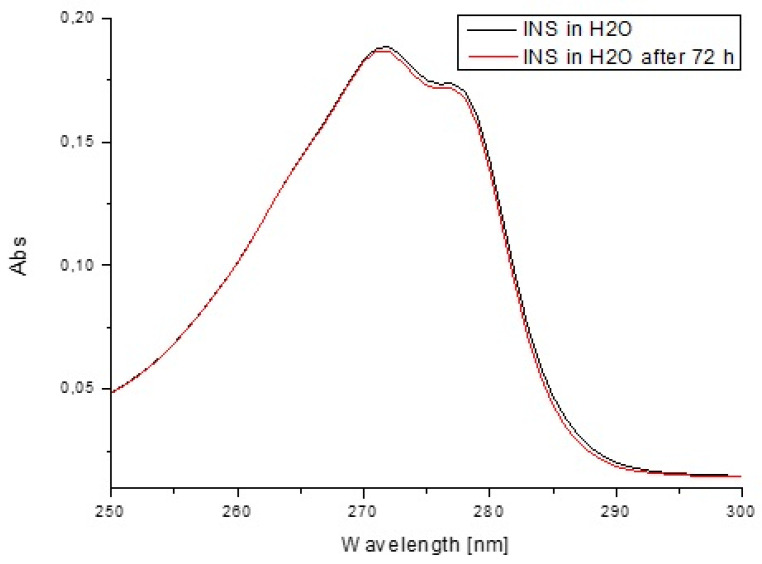
Absorption spectra of insulin at 1.04 × 10^−5^ g/cm^3^ concentration both in H_2_O immediately after sample preparation and after 72 h.

**Figure 16:**
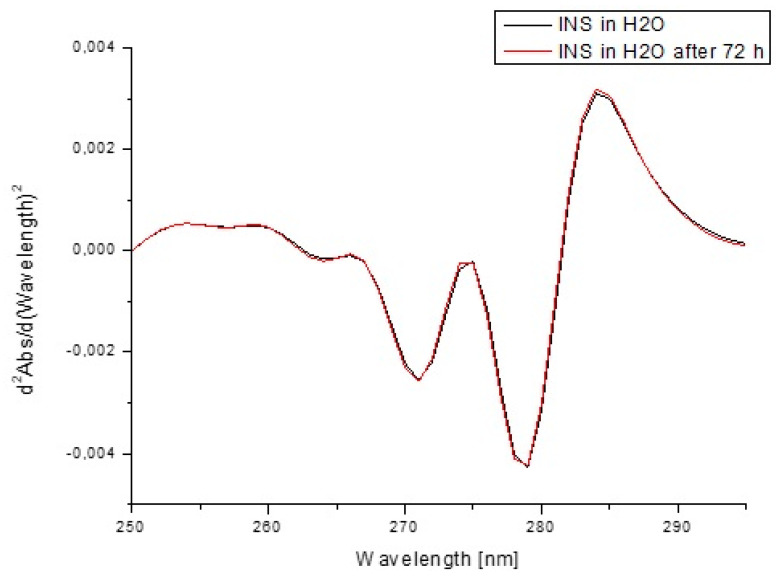
Second derivative absorption spectra of insulin at 1.04 × 10^−5^ g/cm^3^ concentration both in H_2_O immediately after sample preparation and after 72 h.

In order to estimate insulin stability based on the fluorescence intensity (Figure 13) and the absorption values (Figure 14) as well as their second derivatives (Figure 15 and Figure 16), the analysis was carried out for free insulin immediately after sample preparation and after 72 h. All measurements were conducted using the same parameters as for the analysis of insulin in the system with H1–H4 carriers.

## 4. Discussion

Carbopol^®^ Ultrez^TM^ 10 and Carbopol^®^ Ultrez^TM^ 30 are water-soluble polymers that, as a result of ionization, are strongly densified and adopt a gel structure. In terms of chemical structure, they are cross-linked homopolymers of acrylic acid. Adding a base to the Carbopol solution initiates the formation of negative charges along the polymer chain, and their mutual repulsion causes the molecule to expand. Hydrogels based on Carbopol^®^ Ultrez^TM^ 10 and 30 are characterized by inhibition, e.g., absorption of water/aqueous solutions without a significant increase in their volume. Strong intermolecular interactions take place between the polymer chains, which contributes to their high thermal stability. The presence of strong covalent bonds gives them higher chemical and temperature stability compared to conventional ointment bases [55]. Moreover, they show compatibility with many active substances (acidic, basic, and neutral drugs) and high bioadhesives [56,57]. It has a protective effect on the enzymatic degradation of a peptide-like substrate [58]. Zinov’ev et al. [59] concluded in the rat model that Carbopol is a promising carrier for wound-healing preparations in the treatment of necrotic lesions in diabetic foot syndrome. Topical application of a Carbopol-based hydrogel with added antiseptics (poviargol) and nanostructural components accelerates wound healing by 8.4 days, reduces the frequency of suppuration by 23.3%, and exhibits potent bactericidal activity. Carbopol hydrogel incorporating highly skin-permeable growth factors, quercetin, and oxygen carriers accelerate wound healing in the diabetic. The preparation influences the proliferation of keratinocytes and fibroblasts and the chronic wound closure rate [60]. Hui et al. [61] confirmed that the rh-aFGF (recombinant human acidic fibroblast growth factor)-Carbopol hydrogel effect on wound healing in a diabetic rat model was superior to the rh-aFGF solution.

As with Islam et al. [42], we observed that the hydrogels based on Carbopol^®^ Ultrez^TM^ 10 and 30 were shear-thinning fluids characterized by a low degree of thixotropy and the presence of yield stress (Figure 5). The flow curves with increasing and decreasing shear rates were best described by the Herschel–Bulkley model, which was also confirmed by Meng et al. [25,62]. At T = 25 °C, the curves were very close to each other, whereas at T = 32 °C, the hysteresis area increased slightly (H1-INS 10.66 Pa/s vs. 16.68 Pa/s; H2-INS 31.40 Pa/s vs. 39.09 Pa/s). Small areas of the hysteresis loop suggest a quick reconstruction of the hydrogel structure and, consequently, stability during its application. H1-INS was characterized by low yield stress values (T = 25 °C, τ_0_ = 1.6 Pa; T = 32 °C, τ_0_ = 6.2 Pa), whereas for H2-INS, τ_0_ had higher values (T = 25 °C, τ_0_ = 34.8 Pa; T = 32 °C, τ_0_ = 34.4 Pa). Low yield stress values of the H1-INS formulation suggest a lighter texture, easier melting of the hydrogel on the skin surface, and a higher insulin diffusion coefficient at the application site, which was confirmed in the pharmaceutical availability study. A higher yield stress value of the H2-INS formulation indicates its better adhesion [42].

Hydrogels based on methyl cellulose and glycerol ointment are used in the pharmaceutical compounding of dermatological drug products. Methyl cellulose is a semi-synthetic polymer, cellulose methyl ether, containing 27%–32% of methoxy groups. It is characterized by high stability and transparency. After application to the skin, it may leave a polymer film, giving the feeling of pulling [63]. Glycerol ointment is a hydrogel preparation described in the Polish Pharmacopoeia VI [64]. It belongs to water-soluble bases. Wheat starch, which has gelling properties, is a swelling substance in a mixture of glycerol with water. Cellulose and wheat starch are compounds commonly found in nature, therefore, hydrogels based on them were included in the analysis. A significant advantage of methyl cellulose is its ability to bind water in an amount several times greater than the weight of its dry form. Hydrogels based on methyl cellulose are non-toxic, biodegradable, and biocompatible. These features allow their use in several pharmaceutical formulations [65]. These types of hydrogels can potentially have long-term residence in the body because humans lack the cellulose-digesting enzyme [66]. Stalling et al. [67] confirmed that cultured human dermal fibroblasts in the presence of methyl cellulose hydrogels in vitro did not result in significant changes in cell viability after 5 days, indicating the non-cytotoxic of this material. Lan et al. [68] developed hydrogel dressing made of Pluronic F-127 and methyl cellulose for localized and sustained release of siMMP-9 to treat diabetic chronic wounds. Excessive expression of matrix metalloproteinase 9 (MMP-9) impaired the healing of chronic diabetic wounds. The hybrid hydrogel demonstrated high efficacy. In a clinical trial involving 13 subjects, it was confirmed that, by the eighth week after application of the methyl cellulose-based hydrogel, there was complete healing of the venous ulcer [69]. Glycerol is an ingredient used in ointments for burns that may improve skin hydration in diabetic patients [70].

At pH = 4–10, a methyl cellulose-based hydrogel behaved as a pseudoplastic shear-thinning liquid with yield stress. The flow curves were best described by the Herschel–Bulkley model [71,72]. Our research confirmed the above properties of the hydrogel. The yield stress of H3-INS (similarly to H1-INS and H2-INS) increased with an increasing temperature (T = 25 °C, τ_0_ = 4.6 Pa; T = 32 °C, τ_0_ = 14.2 Pa), and its value suggests easy application onto the skin. For the H4-INS hydrogel, the yield stress values were the highest (T = 25°C, τ_0_ = 52.0 Pa; T = 32°C, τ_0_ = 39.6 Pa), therefore, its adhesion to the skin surface is the greatest.

The assessment of formulation texture parameters is an important part of pre-formulation studies aimed at optimizing the formula of semi-solid drug forms. The determination of their value enables us to predict the possibility of using a drug at the site of application and its therapeutic effect. The hardness values of the analyzed formulations did not differ significantly. Adhesiveness, which is compatible with hydrogel bioadhesiveness, was comparable for the individual groups. On the other hand, cohesiveness (>1) confirmed their high consistency and homogeneity. Good quality parameters obtained for Carbopols were probably due to the concentration used (0.5%). Tan et al. stated that the cohesiveness for Carbopol decreased with the increase of its concentration [73].

It was found that Carbopols ensured high pharmaceutical availability of the drug substance [25]. In total, 70% of insulin was released from the Carbopol^®^ Ultrez^TM^ 10 hydrogel matrix, and 65% was released from the Carbopol^®^ Ultrez^TM^ 30 hydrogel within 3 h. In comparison, 75% of insulin was released from the methyl cellulose-based hydrogel after 9 h, and 60% was released from the glycerol ointment after 6 h. The shorter time of insulin release from the H1 and the H2 matrices resulted from their chemical structure. Hydrated, fourth-row amine groups inhibited the penetration of the active substance into the network. After the carrier was applied to the skin, insulin was easily released from its external surface [74]. Moon et al. [75] suggested that the release dynamics and the duration of drug release from the hydrogel depended on the hydrophobicity of the drug, its molecular weight, and the intermolecular interaction between the drug and the polymer. The different insulin release times obtained in this study can be explained by differences in insulin–polymer interactions. Stronger interaction between methyl cellulose and insulin reduced the rate of release of the hormone from the methyl cellulose-based hydrogel. A similar relationship was observed by Park et al. [76] in a study of the effect of methyl cellulose on the drug release behavior of silk fibroin (SF) hydrogel. The presence of methyl cellulose in SF/MC hydrogel decreased the release rate of 5-aminosalicylic acid vs. SF hydrogel. This was due to the hydrophilic interaction between the MC and the drug.

We found no relationship between the viscosity of the analyzed preparations and the amount of insulin release/diffusion rate from the analyzed formulations. Literature data confirm that semi-solid preparations of high viscosity can show both high and low diffusion rates compared to semi-solid preparations of lower viscosity [77]. One of the important problems of topical insulin administration is its short half-life (3–5 min in the blood) and the loss of bioactivity due to the presence of peptidases in the wound environment [78,79]. A solution to this problem may be a prolonged insulin release from the carrier during the treatment of a chronic wound [78].

CD spectra of free insulin in H_2_O and in the presence of hydrogel carriers showed two minima at about 210 and 222 nm (Figure 8), which were typical of α-helix structure, and this phenomenon was in close agreement with spectra obtained by others [80] (Figure 8). The band intensities of INS changed significantly due to the presence of model hydrogels (Table 7). The most significant changes were presented for INS-H1 and INS-H2 systems, while fewer changes, almost identical for INS-H3 and INS-H4 (with lacked changes between far-UV CD spectra of insulin in H3 and H4) were registered. It can be concluded that both H1 and H2 hydrogels slightly influenced INS α-helix and β-sheet contents, while H3 and H4 did not significantly influence the insulin secondary structure elements content. From CD analysis, it can be seen that hydrogels H3 and H4 did not cause the destabilization of the INS secondary structure, which could be clinical importance due to the fact that disorders of the secondary structure of this peptide hormone might have influence on its pharmaceutical availability.

The analysis of insulin fluorescence changes due to the presence of model hydrogels H1–H4 compared to insulin in H_2_O allowed us to monitor INS structural alterations (Figure 9). Data collected in Table 8 show the observed changes in INS fluorescence when comparing the fluorescence intensity of insulin in H_2_O and in the presence of carriers. Due to this phenomenon, it can be assumed that model hydrogels influence the insulin tertiary structure; in order to confirm this phenomenon, the spectral parameters A were calculated. Spectral parameter A ($A=\frac{{\mathrm{Int}}_{290\;\mathrm{nm}}}{{\mathrm{Int}}_{310\;\mathrm{nm}}}$) was used because of its sensitivity to small changes in insulin maximum fluorescence. As in the present study, Maciążek–Jurczyk et al. [81], based on the parameters A as well as FWHM values, analyzed the changes in the tertiary structure of native and oxidized human serum albumin. They observed the decrease in the values of parameters A and FWHM, pointing to the increase in tyrosyl residues and environmental hydrophobicity. Analyzing the spectra parameters A values, it can be concluded that H4 carrier is a factor that could modify the tertiary structure of insulin the most. Studies obtained by Maciążek-Jurczyk et al. [82] also proved that spectral parameters A allowed them to determine the changes in the spatial structure. Similar results were obtained by the analysis of insulin absorbance changes due to the presence of model hydrogels H1–H4 compared to free insulin in H_2_O. The studies of spectra allowed us to monitor INS structural alterations in the tertiary structure (Figure 11), especially in the presence of H1 and H2 hydrogels. H3 and H4 hydrogels decreased, in the same content, the absorbance value of insulin.

The second derivative of fluorescence/absorption spectra is a useful parameter in the assessment of changes occurring in the environment of aromatic amino acids. Appropriate use of second derivative spectroscopy allows one to reduce the interference caused by the presence of the solvent or complete elimination. The obtained second derivative of fluorescence spectra in the wavelength range from 285 nm to 325 nm showed changes within tyrosyl residues. Figure 3 shows the changes in the intensity of fluorescence spectra of the second derivative of INS due to the presence of hydrogel carriers, and the values of $d^{2}\mathrm{Int}/d{\left(\mathrm{Wavelength}\right)}^{2}$ at λ_max_ 295 nm are collected in Table 8. Second derivative spectroscopy allows for better detecting slight spectrum features (for example, shoulders, ripples) and their improvement qualification with very low or insignificant error. Due to these properties, it is possible to accurately determine the changes in the environment around the aromatic amino acid residues [83]. The changes in the second derivative of fluorescence (Figure 10) and absorption spectra (Figure 12) confirmed the influence of model hydrogels, especially H2 and H4, on INS tertiary structure, but the alterations were not significant.

In order to confirm that the changes in the insulin tertiary structure were obtained due to the presence of model H1–H4 hydrogels, the stability of insulin in H_2_0 immediately after sample preparation and after 72 h based on the fluorescence intensity (Figure 13) as well as the absorption spectra (Figure 14) and their second derivatives (Figure 15 and Figure 16) were shown. There being no significant changes (less than 2%) confirmed model hydrogels carriers influenced the insulin secondary and tertiary structures, and the stability of insulin was proved.

Hydrogels are among the optimal carriers of peptides and proteins [84]. Our results suggest that effective treatment of diabetic foot ulcers can be achieved with a methyl cellulose-based insulin preparation (H3-INS). Our pre-formulation studies are promising, and they should be continued in animal models in vivo.

## 5. Conclusions

The obtained results indicate that hydrogels are promising carriers in the treatment of diabetic foot ulcers. The most effective parameters of the effectiveness of the analyzed formulations were obtained for the insulin hydrogel based on methyl cellulose. The base gradually released the active ingredient, reaching 75% after 9 h. The insulin release profile was best described by the Korsmeyer–Peppas model. The hydrogel behaved as a pseudoplastic shear thinning liquid with a yield stress. The value of τ_0_ = 14.2 Pa/32 °C suggests easy spreading of the preparation on the skin, which increases the surface of insulin diffusion. The flow curve was best described by the Herschel–Bulkley model. In the texture analysis, the H3-INS formulation was characterized by lowest stiffness (%R: 74.9%), easier application to the skin (hardness1: 0.040 N/m^2^, T = 32 °C), and high consistency and homogeneity. Based on the circular dichroism (CD) spectra as well as fluorescence and absorption spectroscopy, it can be concluded that not stability but the presence of model hydrogel carriers influences the secondary and the tertiary insulin structures, and these alterations can have a key role in its pharmacological action.

## Figures and Tables

**Table 1:** Prescription composition of hydrogels.

Components	H1	H2	H3	H4
	Content [g]			
Carbopol^®^ Ultrez™ 10	0.5	-	-	-
Carbopol^®^ Ultrez™ 30	-	0.5	-	-
Methyl cellulose	-	-	4	-
Wheat starch	-	-	-	14
Glycerol 86%	-	-	-	73
Ethanol 95%	-	-	-	1
Purified water	up to 100	up to 100	up to 100	12

H1, a hydrogel based on Carbopol^®^ Ultrez™ 10; H2, a hydrogel based on Carbopol^®^ Ultrez™ 30; H3, a hydrogel based on methyl cellulose; H4, a hydrogel based on glycerol ointment.

**Table 2:** Kinetic parameters and fitting of mathematical models to the data of insulin release from selected hydrogels.

Kinetics Models	Parameters	Formula Code			
		H1-INS	H2-INS	H3-INS	H4-INS
Zero Order model	R^2^	0.5976	0.3617	0.4067	0.2224
	*k*_0_ (%min^−1^)	0.424	0.396	0.170	0.142
	AIC	91.84	93.10	253.55	208.88
First Order model	R^2^	0.9425	0.8702	0.8354	0.7288
	*k*_1_ (min^−1^)	0.007	0.007	0.004	0.003
	AIC	68.50	73.99	217.64	183.59
Higuchi model	R^2^	0.9548	0.9564	0.9617	0.9044
	*k_H_* (%min^−1/2^)	5.191	4.883	3.536	2.964
	AIC	65.59	60.89	176.86	158.58
Korsmeyer-Peppas model	R^2^	0.9472	0.9540	0.9929	0.9290
	*k_kp_* (min^−n^)	4.183	5.478	6.351	5.023
	n	0.545	0.476	0.399	0.410
	AIC	69.46	62.39	130.75	152.38

k, release constant; R^2^ adjusted, adjusted coefficient of determination; n, release exponent; AIC, Akaike information criterion.

**Table 3:** Pairwise comparison of the similarity factors *f*1 and *f*2 of the drug release profiles.

Formula Code	*f*1	*f*2	Dissolution Profile
H1-INS vs. H2-INS	7.69	68.29	Similar
H1-INS vs. H3-INS	17.20	46.65	Dissimilar
H1-INS vs. H4-INS	29.20	37.60	Dissimilar
H2-INS vs. H3-INS	15.70	49.60	Dissimilar
H2-INS vs. H4-INS	29.31	40.49	Dissimilar
H3-INS vs. H4-INS	19.91	49.38	Dissimilar

**Table 4:** Apparent viscosity values at different shear rates ± SD.

Formula Code	η (30 s^−1^) (mPa × s)	η (50 s^−1^) (mPa × s)	η (100 s^−1^) (mPa × s)
	25 ± 1 °C		
H1-INS	4447 ± 1378	2920 ± 272.1	2059 ± 623.9
H2-INS	3826 ± 133.8	3195 ± 310.2	2023 ± 41.6
H3-INS	4456 ± 2285	3621 ± 1472	1921 ± 586.6
H4-INS	4310 ± 2574	3263 ± 999	2465 ± 743.3
	**32 ± 1 °C**		
H1-INS	4003 ± 1252	2901 ± 893.9	1917 ± 584.3
H2-INS	3678 ± 459.3	2692 ± 834.8	1962 ± 596
H3-INS	4037 ± 594.9	2995 ± 923.1	1894 ± 558.6
H4-INS	3707 ± 867.2	3146 ± 963.7	1898 ± 578

**Table 5:** The results obtained from mathematical modeling of rheogram.

Formula Code	Herschel–Bulkley				Ostwald-de Waele			Bingham		Casson	
	τ_0_	n	K	R^2^	n	K	R^2^	τ_0_	R^2^	τ_0_	R^2^
	25 ± 1 °C										
H1-INS	1.6	0.520	18.50	0.994	0.520	18.5	0.993	58.464	0.970	26.713	0.985
H2-INS	34.8	0.681	8.54	0.997	0.480	24.5	0.989	65.148	0.992	32.777	0.995
H3-INS	4.6	0.783	9.40	0.998	0.754	10.8	0.997	38.984	0.996	8.881	0.997
H4-INS	52.0	0.990	2.86	0.993	0.604	18.9	0.968	48.746	0.990	18.975	0.984
	**32 ± 1 °C**										
H1-INS	6.2	0.523	19.20	0.999	0.494	22.4	0.998	65.985	0.977	31.906	0.992
H2-INS	34.4	0.719	7.70	0.995	0.513	22.4	0.987	63.299	0.991	29.541	0.993
H3-INS	14.2	0.836	6.85	0.999	0.737	11.1	0.997	39.174	0.993	9.343	0.997
H4-INS	39.6	0.940	3.31	0.998	0.615	15.8	0.980	43.424	0.995	16.133	0.993

τ_0_: yield stress (Pa); n: flow behavior index; Κ: consistency index; R^2^: regression coefficient.

**Table 6:** Textural parameter values of hydrogels (mean ± SD, *n* = 3).

Formula Code	Relaxation (%)	Hardness1 (N/m^2^)	Hardness2 (N/m^2^)	Cohesiveness	Adhesiveness (mJ)	Elasticity
	25 ± 1 °C					
H1-INS	54.2% ± 1.02 ^a^	0.061 ± 0.02 ^a^	0.069 ± 0.04 ^b^	1.087 ± 0.02 ^a^	0.3 ± 0.01 ^a^	0.959 ± 0.06 ^c^
H2-INS	58.0% ± 0.58 ^a^	0.061 ± 0.01 ^a^	0.074 ± 0.02 ^b^	1.638 ± 0.04 ^b^	0.3 ± 0.02 ^a^	0.696 ± 0.03 ^b^
H3-INS	72.7% ± 0.67 ^b^	0.042 ± 0.03 ^b^	0.054 ± 0.03 ^a^	2.820 ± 0.02 ^d^	0.1 ± 0.01 ^a^	0.499 ± 0.04 ^a^
H4-INS	56.5% ± 0.55 ^a^	0.060 ± 0.01 ^a^	0.063 ± 0.02 ^b^	2.080 ± 0.06 ^c^	0.3 ± 0.02 ^a^	0.555 ± 0.02 ^a^
	**32 ± 1 °C**					
H1-INS	50.4% ± 1.05 ^b^	0.060 ± 0.01 ^a^	0.068 ± 0.02 ^b^	1.119 ± 0.06 ^a^	0.3 ± 0.02 ^a^	0.996 ± 0.05 ^c^
H2-INS	42.0% ± 0.34 ^a^	0.063 ± 0.02 ^a^	0.071 ± 0.01 ^b^	1.281 ± 0.03 ^a^	0.3 ± 0.02 ^a^	0.823 ± 0.04 ^b^
H3-INS	74.9% ± 1.00 ^d^	0.040 ± 0.02 ^b^	0.047 ± 0.02 ^a^	3.468 ± 0.04 ^b^	0.2 ± 0.01 ^a^	0.426 ± 0.03 ^a^
H4-INS	63.2% ± 0.95 ^c^	0.062 ± 0.03 ^a^	0.067 ± 0.03 ^b^	1.292 ± 0.05 ^a^	0.2 ± 0.01 ^a^	0.836 ± 0.04 ^b^

^a,b,c,d^ mean values in the columns (at T = 25 ± 1 °C and T = 32 ± 1 °C, respectively) marked with letters are statistically significantly different (*p* < 0.05).

**Table 7:** The percentage (%) content of the secondary structure elements of insulin based on Reed’s reference model.

Form of Insulin	(θ)_mre_ (deg·cm^2^·dmol^−1^)	(θ)_mre_ (deg·cm^2^·dmol^−1^)	% α-Helix	% β-Sheet	% Random
INS in H_2_O	at 209.4 nm −12529.96	at 219.6 nm −10377.76	44.8	47.0	8.2
INS in H1	at 210 nm −8808.33	at 223.8 nm −8335.91	26.6	52.5	20.9
INS in H2	at 213.0 nm −4742.83	at 224.2 nm −5360.92	28.6	55.0	16.5
INS in H3	at 209.6 nm −13245.26	at 222.0 nm −11572.65	52.1	47.9	0
INS in H4	at 209.8 nm −13035.82	at 223.2 nm −11531.60	44.9	48.3	6.8

**Table 8:** Insulin fluorescence parameters both in H_2_O and in the presence of model hydrogels H1–H4 at λ_ex_ 276 nm.

Form of Insulin	Fluorescence Intensity (Int) at λ_max_	$A=\frac{Int_{290\;nm}}{Int_{310\;nm}}$	$d^{2}Int/d{\left(Wavelength\right)}^{2}$ at λ_max_ (295 nm)
INS in H_2_O	652.175 λ_max_ 300 nm	0.916	−4.51215
INS in H1	648.642 λ_max_ 299 nm	0.910	−4.46256
INS in H2	631.685 λ_max_ 299 nm	0.906	−4.31653
INS in H3	661.318 λ_max_ 299 nm	0.908	−4.54568
INS in H4	644.559 λ_max_ 299 nm	0.899	−4.39367

## Data Availability

Samples of the formulations are available from the authors.

## References

[1] Gerich J.E. (2002). Is Reduced First-Phase Insulin Release the Earliest Detectable Abnormality in Individuals Destined to Develop Type 2 Diabetes?. Diabetes.

[2] Rho H.S., Veltkamp H.W., Hanke A.T., Ottens M., Breukers C., Habibović P., Gardeniers H. (2020). Systematic Investigation of Insulin Fibrillation on a Chip. Molecules.

[3] Chang S.G., Choi K.D., Jang S.H., Shin H.C. (2003). Role of disulfide bonds in the structure and activity of human insulin. Mol. Cells.

[4] Rachdaoui N. (2020). Insulin: The Friend and the Foe in the Development of Type 2 Diabetes Mellitus. Int. J. Mol. Sci..

[5] Haeusler R.A., McGraw T.E., Accili D. (2018). Biochemical and cellular properties of insulin receptor signalling. Nat. Rev. Mol. Cell. Biol..

[6] Quitério M., Simões S., Ascenso A., Carvalheiro M., Leandro A.P., Correia I., Viana A.S., Faísca P., Ascensão L., Molpeceres J. (2021). Development of a Topical Insulin Polymeric Nanoformulation for Skin Burn Regeneration: An Experimental Approach. Int. J. Mol. Sci..

[7] Abdelkader D.H., Osman M.A., Elgizawy S.A., Faheem A.M., McCarron P.A. (2016). The Role of Insulin in Wound Healing Process: Mechanism of Action and Pharmaceutical Applications. J. Anal. Pharm. Res..

[8] Shukla S.K., Sharma A.K., Gupta V., Yashavarddhan M.H. (2019). Pharmacological control of inflammation in wound healing. J. Tissue Viability.

[9] Pendsey S.P. (2010). Understanding diabetic foot. Int. J. Diabetes Dev. Ctries.

[10] Whitaker I.S., Twine C., Whitaker M.J., Welck M., Brown C.S., Shandall A. (2007). Larval therapy from antiquity to the present day: Mechanisms of action, clinical applications and future potential. Postgrad. Med. J..

[11] Dehkordi A.N., Babaheydari F.M., Chehelgerdi M., Dehkordi S.R. (2019). Skin tissue engineering: Wound healing based on stem-cell-based therapeutic strategies. Stem. Cell. Res. Ther..

[12] Abdelkader D.H., Osman M.A., El-Gizawy S.A., Hawthorne S.J., Faheem A.M., McCarron P.A. (2018). Effect of poly(ethylene glycol) on insulin stability and cutaneous cell proliferation in vitro following cytoplasmic delivery of insulin-loaded nanoparticulate carriers-A potential topical wound management approach. Eur. J. Pharm. Sci..

[13] Goenka G., Athavale V.S., Nirhale D.S., Deshpande N., Agrawal K., Calcuttawala M. (2014). Role of topical use of insulin in healing of chronic ulcer. Med. J. DY Patil. Univ..

[14] Besson J.C.F., Hernandes L., Campos J.M., Morikawa K.A., Bersani-Amado C.A., Matioli G. (2017). Insulin complexed with cyclodextrins stimulates epithelialization and neovascularization of skin wound healing in rats. Injury.

[15] Lima M.H., Saad M. (2007). Processo de Obtenção de Uma Preparação Farmacêutica, Preparação Farmacêutica e Seu Uso Como Medicamento para Lesões Ulceradas. BRPI0705370 B1.

[16] Lima M.H.M., Caricilli A.M., de Abreu L.L., Araújo E.P., Pelegrinelli F.F., Thirone A.C.P., Tsukumo D.M., Pessoa A.F.M., dos Santos M.F., de Moraes M.A. (2012). Topical Insulin Accelerates Wound Healing in Diabetes by Enhancing the AKT and ERK Pathways: A Double-Blind Placebo-Controlled Clinical Trial. PLoS ONE.

[17] Zhao L., Niu L., Liang H., Tan H., Liu C., Zhu F. (2017). pH and glucose dual-responsive injectable hydrogels with insulin and fibroblasts as bioactive dressings for diabetic wound healing. ACS Appl. Mater. Interfaces.

[18] Sridharan K., Sivaramakrishnan G. (2017). Efficacy of topical insulin in wound healing: A preliminary systematic review and meta-analysis of randomized controlled trials. Wound Repair Regen..

[19] Lim J.Y.C., Goh S.S., Loh X.J. (2020). Bottom-Up Engineering of Responsive Hydrogel Materials for Molecular Detection and Biosensing. ACS Mater. Lett..

[20] Ravaine V., Ancla C., Catargi B. (2008). Chemically controlled closed-loop insulin delivery. J. Control. Release.

[21] Matsumoto A., Tanaka M., Matsumoto H., Ochi K., Moro-Oka Y., Kuwata H., Yamada H., Shirakawa I., Miyazawa T., Ishii H. (2017). Synthetic “smart gel” provides glucose-responsive insulin delivery in diabetic mice. Sci. Adv..

[22] Doostmohammadi M., Ameri A., Mohammadinejad R., Dehghannoudeh N., Banat I.M., Ohadi M., Dehghannoudeh G. (2019). (2019). Hydrogels For Peptide Hormones Delivery: Therapeutic And Tissue Engineering Applications. Drug. Des. Devel. Ther..

[23] Jacyna B., Maciejewski B., Sznitowska M. (2020). Hydrożele w recepturze aptecznej leków dermatologicznych. Farm. Pol..

[24] Polskie Towarzystwo Farmaceutyczne (2017). Polish Pharmacopoeia XI.

[25] Meng Z., Ancey C. (2019). The effects of slide cohesion on impulse-wave formation. Exp. Fluids.

[26] Timofeev V.I., Baidus A.N., Kislitsyn Y.A., Kuranova I.P. Structure of Human Insulin. PDB Id: 3E7Y. To Be Published. https://www.rcsb.org/structure/3e7y.

[27] (1998). Quality of biotechnological products: Stability testing of biotechnological/biological products. Annex to the ICH Harmonised Tripartite Guideline for the Stability Testing of New Drug Substances and Products. Dev. Biol. Stand..

[28] U.S. Pharmacopeial Convention (2009). United States Pharmacopeia, 32.

[29] Cardia M.C., Carta A.R., Caboni P., Maccioni A.M., Erbì S., Boi L., Meloni M.C., Lai F., Sinico C. (2019). Trimethyl Chitosan Hydrogel Nanoparticles for Progesterone Delivery in Neurodegenerative Disorders. Pharmaceutics.

[30] Shehata T.M., Nair A.B., Al-Dhubiab B.E., Shah J., Jacob S., Alhaider I.A., Attimarad M., Elsewedy H.S., Ibrahim M.M. (2020). Vesicular Emulgel Based System for Transdermal Delivery of Insulin: Factorial Design and in Vivo Evaluation. Appl. Sci..

[31] Mumuni A.M., Calister E.U., Aminu N., Franklin C.K., Musiliu Oluseun A., Usman M., Abdulmumuni B., James Y.O., Ofokansi C.K., Anthony A.A. (2020). Mucin-Grafted Polyethylene Glycol Microparticles Enable Oral Insulin Delivery for Improving Diabetic Treatment. Appl. Sci..

[32] Zhang Y., Huo M., Zhou J., Zou A., Li W., Yao C., Xie S. (2010). DDSolver: An add-in program for modeling and comparison of drug dissolution profiles. AAPS J..

[33] Verrez-Bagnis V., Bouchet B., Gallant D.J. (1993). Relationship between the starch granule structure and the textural properties of heat–induced surimi gel. Food Struct..

[34] Szczesniak A.S. (2002). Texture is a sensory property. Food Qual. Pref..

[35] Siemiradzka W., Dolińska B., Ryszka F. (2020). Development and Study of Semi-Solid Preparations Containing the Model Substance Corticotropin (ACTH): Convenience Application in Neurodegenerative Diseases. Molecules.

[36] Siemiradzka W., Dolińska B., Ryszka F. (2020). Influence of Concentration on Release and Permeation Process of Model Peptide Substance-Corticotropin-From Semisolid Formulations. Molecules.

[37] Kelly S.M., Jess T.J., Price N.C. (2005). How to study proteins by circular dichroism. Biochim. Biophys. Acta.

[38] Saifullah M., Yusof Y.A., Chin N.L., Aziz M.G., Mohammed M.A.P., Aziz N.A. (2016). Dissolution profiling and its comparison of natural fruit powder effervescent tablets. J. Food Eng..

[39] Bom S., Santos C., Barros R., Martins A.M., Paradiso P., Cláudio R., Pinto P.C., Ribeiro H.M., Marto J. (2020). Effects of Starch Incorporation on the Physicochemical Properties and Release Kinetics of Alginate-Based 3D Hydrogel Patches for Topical Delivery. Pharmaceutics.

[40] Destruel P.L., Zeng N., Brignole-Baudouin F., Douat S., Seguin J., Olivier E., Dutot M., Rat P., Dufaÿ S., Dufaÿ-Wojcicki A. (2020). In Situ Gelling Ophthalmic Drug Delivery System for the Optimization of Diagnostic and Preoperative Mydriasis: In Vitro Drug Release, Cytotoxicity and Mydriasis Pharmacodynamics. Pharmaceutics.

[41] Kim J.Y., Song J.Y., Lee E.J., Park S.K. (2003). Rheological properties and microstructures of Carbopol gel network system. Colloid Polym. Sci..

[42] Islam M.T., Rodríguez-Hornedo N., Ciotti S., Ackermann C. (2004). Rheological characterization of topical carbomer gels neutralized to different pH. Pharm. Res..

[43] Kieweg S.L., Geonnotti A.R., Katz D.F. (2004). Gravity-induced coating flows of vaginal gel formulations: In vitro experimental analysis. J. Pharm. Sci..

[44] Isaac V.L.B., Moraes J.D.D., Chiari B.G., Guglielmi D.A.S., Cefali L.C., Rissi N.C., Corrêa M.A. (2013). Determination of the real influence of the addition of four thickening agents in creams using rheological measurements. J. Dispers. Sci. Technol..

[45] Nazaré A.C., de Faria C.M., Chiari B.G., Petrônio M.S., Regasini L.O., Silva D.H., Corrêa M.A., Isaac V.L., da Fonseca L.M., Ximenes V.F. (2014). Ethyl ferulate, a component with anti-inflammatory properties for emulsion-based creams. Molecules.

[46] Lee C.H., Moturi V., Lee Y. (2009). Thixotropic property in pharmaceutical formulations. J. Control. Release.

[47] Carvalho F.C., Calixto G., Hatakeyama I.N., Luz G.M., Gremião M.P., Chorilli M. (2013). Rheological, mechanical, and bioadhesive behavior of hydrogels to optimize skin delivery systems. Drug Dev. Ind. Pharm..

[48] Bansal K., Rawat M.K., Jain A., Rajput A., Chaturvedi T.P., Singh S. (2009). Development of satranidazole mucoadhesive gel for the treatment of periodontitis. AAPS Pharmscitech.

[49] Can A.S., Erdal M.S., Güngör S., Özsoy Y. (2013). Optimization and Characterization of Chitosan Films for Transdermal Delivery of Ondansetron. Molecules.

[50] Shaw G.S., Uvanesh K., Gautham S.N., Singh V., Pramanik K., Banerjee I., Kumar N., Pal K. (2015). Development and characterization of gelatin-tamarind gum/carboxymethyl tamarind gum based phase-separated hydrogels: A comparative study. Des. Monomers Polym..

[51] Singh V.K., Yadav I., Kulanthaivel S., Roy B., Giri S., Maiti T., Banerjee I., Pal K. (2016). Groundnut oil based emulsion gels for passive and iontophoretic delivery of therapeutics. Des. Monomers Polym..

[52] Xia H., Ren M., Zou Y., Qin S., Zeng C. (2020). Novel Biocompatible Polysaccharide-Based Eutectogels with Tunable Rheological, Thermal, and Mechanical Properties: The Role of Water. Molecules.

[53] Couto J., Figueirinha A., Batista M.T., Paranhos A., Nunes C., Gonçalves L.M., Marto J., Fitas M., Pinto P., Ribeiro H.M. (2020). Fragaria vesca L. Extract: A Promising Cosmetic Ingredient with Antioxidant Properties. Antioxidants.

[54] Li Y., Jia B., Wand H., Li N., Chen G., Lin Y., Gao W. (2013). The interaction of 2-mercaptobenzimidazole with human serum albumin as determined by spectroscopy, atomic force microscopy and molecular modeling. Colloids Surf..

[55] Tal-Figiel B., Figiel W., Kwiecień M. (2013). Pomiary oscylacyjne leczniczych układów żelowych. Inz. Aparatura Chem..

[56] Hurler J., Engesland A., Poorahmary Kermany B., Škalko-Basnet N. (2012). Improved texture analysis for hydrogel characterization: Gel cohesiveness, adhesiveness, and hardness. J. Appl. Polym. Sci..

[57] Suhail M., Wu P.-C., Minhas M.U. (2020). Using Carbomer-Based Hydrogels for Control the Release Rate of Diclofenac Sodium: Preparation and In Vitro Evaluation. Pharmaceuticals.

[58] Vaidya A.P., Wigent R.J., Moore J.C., Schwartz J.B. (2007). Protective effect of Carbopol on enzymatic degradation of a peptide-like substrate. I: Effect of various concentrations and grades of Carbopol and other reaction variables on trypsin activity. Pharm. Dev. Technol..

[59] Zinov’ev E.V., Ivakhniuk G.K., Dadaian K.A., Lagvilava T.O. (2014). [Wound-healing effect of carbopol hydrogels in rats with alloxan diabetes model]. Eksp. Klin. Farmakol..

[60] Jee J.P., Pangeni R., Jha S.K., Byun Y., Park J.W. (2019). Preparation and in vivo evaluation of a topical hydrogel system incorporating highly skin-permeable growth factors, quercetin, and oxygen carriers for enhanced diabetic wound-healing therapy. Int. J. Nanomed..

[61] Hui Q., Zhang L., Yang X., Yu B., Huang Z., Pang S., Zhou Q., Yang R., Li W., Hu L. (2018). Higher Biostability of rh-aFGF-Carbomer 940 Hydrogel and Its Effect on Wound Healing in a Diabetic Rat Model. ACS Biomater. Sci. Eng..

[62] Meng Z., Hu Y., Ancey C. (2020). Using a Data Driven Approach to Predict Waves Generated by Gravity Driven Mass Flows. Water.

[63] Jiménez M.M., Fresno M.J., Ramírez A. (2005). The influence of cosolvent polarity on the flow properties of hydroalcoholic gels: Empirical models. Chem. Pharm. Bull. (Tokyo).

[64] Państwowy Zakład Wydawnictw Lekarskich (2002). Polish Pharmacopoeia VI.

[65] Peng Z., Chen F. (2010). Synthesis and properties of temperature-sensitive hydrogel based on hydroxyethyl cellulose. Int. J. Polym. Mater..

[66] Gold G.T., Varma D.M., Taub P.J., Nicoll S.B. (2015). Development of crosslinked methylcellulose hydrogels for soft tissue augmentation using an ammonium persulfate-ascorbic acid redox system. Carbohydr. Polym..

[67] Stalling S.S., Akintoye S.O., Nicoll S.B. (2009). Development of photocrosslinked methylcellulose hydrogels for soft tissue reconstruction. Acta Biomater..

[68] Lan B., Zhang L., Yang L., Wu J., Li N., Pan C., Wang X., Zeng L., Yan L., Yang C. (2021). Sustained delivery of MMP-9 siRNA via thermosensitive hydrogel accelerates diabetic wound healing. J. Nanobiotechnol..

[69] Naomi R., Fauzi M.B. (2020). Cellulose/Collagen Dressings for Diabetic Foot Ulcer: A Review. Pharmaceutics.

[70] Federici A., Federici G., Milani M. (2015). Use of a urea, arginine and carnosine cream versus a standard emollient glycerol cream for treatment of severe xerosis of the feet in patients with type 2 diabetes: A randomized, 8 month, assessor-blinded, controlled trial. Curr. Med. Res. Opin..

[71] Jaworska M., Vogt O. (2013). Pochodne sorbitolu i celulozy jako czynniki żelujące. Chemik.

[72] Kulawik-Pióro A., Pogorzelska K. (2015). Rola pochodnych celulozy w wytwarzaniu żeli kosmetycznych. Inz. Ap. Chem..

[73] Tan Y.T.F., Peh K.K., Al-Hambali O. (2000). Effect of carbopol and polyvinylpyrrolidone on the mechanical, rheological, and release properties of bioadhesive polyethylene glycol gels. AAPS Pharmscitech.

[74] Kołodziejczyk M.K., Kołodziejska J. (2015). Niskolepki hydrożel ma bazie karbopolu jako optymalna forma leku z ketoprofenem. eReumatol News.

[75] Moon H.J., Park M.H., Joo M.K., Jeong B. (2012). Temperature-responsive compounds as in situ gelling biomedical materials. Chem. Soc. Rev..

[76] Park C.H., Jeong L., Cho D., Kwon O.H., Park W.H. (2013). Effect of methylcellulose on the formation and drug release behavior of silk fibroin hydrogel. Carbohydr. Polym..

[77] Bao Q., Burgess D.J. (2018). Perspectives on Physicochemical and In Vitro Profiling of Ophthalmic Ointments. Pharm. Res..

[78] Li W., Gao F., Kan J., Deng J., Wang B., Hao S. (2019). Synthesis and fabrication of a keratin-conjugated insulin hydrogel for the enhancement of wound healing. Colloids Surf. B.

[79] Moura L.I., Dias A.M., Leal E.C., Carvalho L., de Sousa H.C., Carvalho E. (2014). Chitosan-based dressings loaded with neurotensin—An efficient strategy to improve early diabetic wound healing. Acta Biomater..

[80] Xu Y., Zhang X., Zhang Y., Ye J., Wang H.L., Xia X., Liu Y. (2018). Mechanisms of deformable nanovesicles based on insulin-phospholipid complex for enhancing buccal delivery of insulin. Int. J. Nanomed..

[81] Maciążek–Jurczyk M., Morak–Młodawska B., Jeleń M., Kopeć W., Szkudlarek A., Owczarzy A., Kulig K., Rogóż W., Pożycka J. (2021). The Influence of Oxidative Stress on Serum Albumin Structure as a Carrier of Selected Diazaphenothiazine with Potential Anticancer Activity. Pharmaceuticals.

[82] Maciążek-Jurczyk M., Janas K., Pożycka J., Szkudlarek A., Rogóż W., Owczarzy A., Kulig K. (2020). Human Serum Albumin Aggregation/Fibrillation and its Abilities to Drugs Binding. Molecules..

[83] Balestrieri C., Colonna G., Giovane A., Irace G., Servillo L. (1978). Second–derivative Spectroscopy of Proteins. A Method for the Quantitative Determination of Aromatic Aminoacids in Proteins. Eur. J. Biochem..

[84] Raza F., Zafar H., Zhu Y., Ren Y., Ullah A., Khan A.U., He X., Han H., Aquib M., Boakye-Yiadom K.O. (2018). A Review on Recent Advances in Stabilizing Peptides/Proteins upon Fabrication in Hydrogels from Biodegradable Polymers. Pharmaceutics.

